# Substance use and associated factors among high school students in Northwest Ethiopia

**DOI:** 10.11604/pamj.2023.44.162.35168

**Published:** 2023-04-06

**Authors:** Mamaru Melkam, Tesfaye Segon, Girum Nakie, Goshu Nenko, Demeke Demilew

**Affiliations:** 1 Department of Psychiatry, College of Medicine and Health Sciences, University of Gondar, Gondar, Ethiopia,; 2 Department of Psychiatry, College of Medicine and Health Sciences, Metu University, Metu, Ethiopia

**Keywords:** Adolescence, prevalence, students, substance use, Ethiopia

## Abstract

**Introduction:**

substance abuse is the use of a drug that affects both the function and the structure of the brain by altering the activity neurotransmitters of particular pathways in the central nervous system that affect the mood, perception, and consciousness of the person.

**Methods:**

a cross-sectional study design was employed among 422 high school students. Study participants were selected by simple random sampling from all five high school students. A self-reported questionnaire was used that included alcohol, smoking, and substance involvement screening test, Oslo-3 social support, and other instruments. Data were checked, coded, and entered into Epi-Data version 4.6.2 then exported to the statistical package for social sciences version 20 for analysis. Bi-variable and multi-variable logistic regression analyses were used to identify factors associated with substance use. Adjusted odds ratios with 95% confidence intervals were determined and predictors with a p-value of <0.05 were counted as significantly associated.

**Results:**

a total of 406 students have participated with an overall response rate of 96.2% (n=406). Of the participants 235 were female and 171 were male. The mean age was 17.51 with a standard deviation of ±1.42. The prevalence of substance use among study participants was 52.5%. Being male aOR: 1.77, 95% CI 1.11-2.77, family history of substance use aOR: 3.07, 95% CI 1.57-6.01, and having close friends´ pressure aOR: 5.77, 95% CI 2.39-13.89 were significantly associated with substances use among high school students.

**Conclusion:**

the prevalence of substance use among high school students was high. Being male, family history of substance use, and having peer pressures were strongly associated with substance use.

## Introduction

Substances use become a concern of the people increasing major public health problems and affecting the socio-economic status worldwide [1]. Adolescence is a development stage estimated to have an addiction to the consumption of high amounts of substances the related problem needs appropriate intervention in the health care system [2]. World Health Organization (WHO) showed around 2 billion people worldwide use alcoholic beverages and around 76.3 million people are affected by alcohol-related problems. The global burden of alcohol consumption has a high impact on health, both in the case of morbidity and mortality which are considered in most parts of the world [3].

An estimated 4% of deaths worldwide are caused by harmful alcohol use, which kills 2.5 million people yearly. Of all male deaths, 6.2% are due to alcohol use, compared to 1.1% of female deaths worldwide. Annually, 320,000 adolescents died from the use of alcohol, and 9% of all deaths were reported from this age group globally [4]. The key underlying illnesses that ultimately lead to the worldwide burden of disease associated with chronic substance use are disorders caused by dependence on psychoactive substances [5]. The prevalence of substance use among students in Ethiopia was 34.8 and 47.9% [6-10]. The use of psychoactive substances among youths inflates the bio-psychological risk of addictive properties together with peer pressure [11]. Recently, the conception of substances particularly alcohol, khat, and tobacco becomes dramatically increased from time to time especially in developing countries [12].

The use of substances by students can lead to harmful effects that decrease academic performance and increase the risk of being infected by HIV and other sexually transmitted infections after intoxication [6,9]. The use of alcohol, especially in large doses and when combined with other drugs like khat and tobacco, poses a serious risk to the lives of many people [13]. The use of drugs by one's parents and close friends, one's gender, one's religious beliefs, and one's lack of trust in others was associated with substance use in several studies among adolescents [6,9,14]. The burden of substance use emanated from different sources including parents, school teachers, and a policeman with wide media coverage due to related crime [15,16]. Use of substances especially stimulant increasingly used for reading among high school students is high in Ethiopia. Our study was conducted to determine the prevalence of substance use and identify factors that influenced the behavior of students in Northwest Ethiopia.

## Methods

**Study design and period:** an institutional-based cross-sectional study was employed from May 5^th^ to May 30^th^, 2021.

**Study setting:** the study was conducted in Debre Markos towns in Amhara Regional State, the administrative center of the East Gojam zone. It is located 265 kilometers to the Northwest of Addis Ababa, the capital city of Ethiopia. Debre Markos town has a total of 113,101 populations, and 60,425 of the population are women. There are seven kebeles in the town and it has only one referral hospital [17]. There have been no privatized high schools in this town, only 5 public high schools. For the calendar year 2021, 8,071 students were registered for classes 9 through 12.

**Study population:** all high school students who were engaged in their education in high school were a source of population. High school students who were present at the time of data collection in the town were considered as study populations.

**Eligibility criteria:** high school students found in class at the time of data collection were included in the study but students who have been acutely sick at school at the time of data collection were excluded.

**Figure 1:**
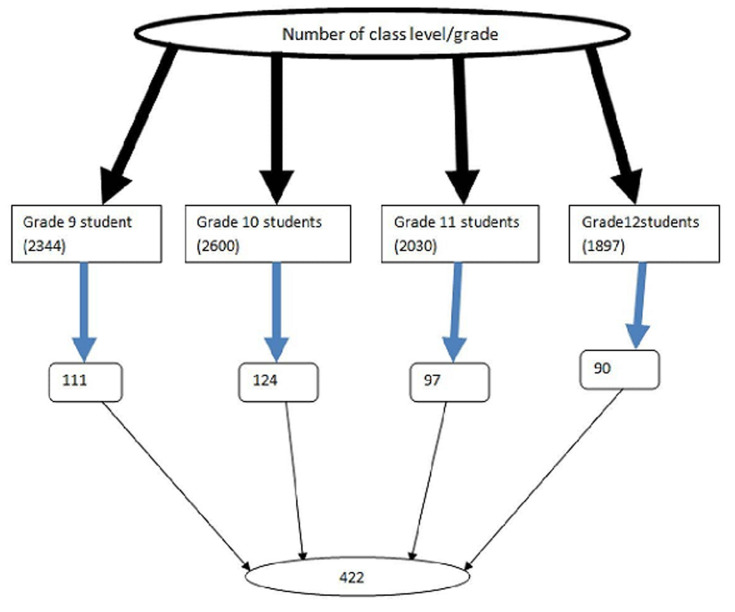
the method for determining sample size from high school students in Northwest Ethiopia, in the year 2021

**Sample size determination and sampling procedure:** the minimum adequate sample size was estimated by single population proportion and two population proportion formulas. The following assumptions were considered prevalence p=47.9% taken from the previous similar study which was done in Woreta town among high school students [6], 95% CI, a margin of error of 5%, and 10% non-response rate. The final calculated sample size was 422. Simple random sampling was performed after the study respondents were proportionally assigned to each stratum or class level. Specifically, computer-generated simple random sampling was used to recruit students. The selected students out of each school's grade level were brought into one room and administered the questions (Figure 1).

**Operational definition:** current substance use: using at least one substance for non-medical purposes within the last 3 months including alcohol, khat, and tobacco. Ever (lifetime) use of a substance: using at least one of any specific substance for a non-medical purpose at least once in a lifetime like alcohol, khat, and tobacco [18]. Common mental disorders: self-reported questioners were used to assess students for the presence of mental illnesses using a 20-item questionnaire [19]. Social support: the assistance of friends, family, and other individuals during times of need might include financial, social, and psychological components [20]. Physical exercises: running, brisk walking, riding, dancing, and playing sports for at least 30 minutes each day, at least five times a week [21]. Known chronic medical illness: the existence of a known chronic illness like diabetes, epilepsy, HIV/AIDS, and others. Physical and non-physical sexual abuse: the act of engaging in sexual activity with kids without their will.

**Data collection procedure and tool:** five-part identity surveys were used to collect data from five high schools; the first section of the questionnaire was about socio-demographics. Alcohol, smoking, and substance involvement screening test (ASSIST), a highly-validated instrument created by the WHO, was adapted for the behavioral variables section of the questionnaire, which included present and ever-use substances as secondary sections [18]. A single question from the global school-based health survey (GSHS) questionnaire created by the WHO and the center for disease prevention and control was used to assess each participant's history of physical activity [21]. As the third section self-reported the questionnaire with 20 yes-or-no questions and 8 as the cut-off point [19]. Lifetime exposure to sexual abuse was included in the questionnaire's fourth section, which assessed psychological issues, including sexual abuse. These questions were taken from the International Society for the Prevention of Child Abuse and Neglect (ISPCAN) child abuse screening tool for children [22] and the Oslo-Social Support Scale [20]. The clinical factors, such as family exposure to mental illness, (do you have any relatives who use drugs or committed suicide or who have a persistent major mental illness) with “yes” or “no” possible answers [23]. The other clinical component was a known chronic medical condition yes-or-no question.

**Data quality control:** the questionnaire was written in English, translated into the Amharic language, and then back to English. One week before data collection, a pretest was administered to (5%) of the total sample size in Fnote Selam town to check the validity and reliability of the instrument, with a Cronbach's alpha of 0.75. After receiving training three bachelor of science (BSC) psychiatry professionals gathered data under two experienced BSC psychiatry supervisors. All data collectors´ periods were observed by supervisors to discuss any issues that happened during data collection time. The collected data were examined and checked for completeness before being entered. For data submitted into Epi-Data version 4.2.0, a template for data entry was created.

**Statistical analysis:** the data were validated, coded, and entered into Epi-Data version 4.6.2 before being exported to SPSS version 20. Descriptive statistics of socio-demographic and other variables were done. The relationship between each variable with outcome variables was determined using bi-variable logistic regression analysis. The multivariable logistic regression model included all variables with a p-value < 0.20 from the bivariate logistic regression analysis. AOR with 95% CI was calculated when P-values of <0.05 was deemed statistically significant. The response was described in tables, figures, and charts using frequency and summary statistics like mean and percentage. The model's fitness was checked by Hosmer and Lemeshow test, which yields a 60.4%.

**Ethical consideration:** before data collection ethical approval was acquired from the University of Gondar's Ethical Review Board with 427/2021. Before the data collection period, written informed consent and assent were obtained from the students and their parents, respectively. Students were made aware of the study and assured it would not have a detrimental effect on their lives. Participants were made aware that they can decline the study's invitation at any moment and confidentiality was maintained. Students were also informed that they might approach the data collectors to be referred to a hospital for further evaluations. Participants were situated far enough apart to ensure privacy so they could complete.

## Results

**Socio-demographic characteristics of the respondent:** from 422 study participants, 406 high school students were included with a response rate of 96.2%. Among the respondents 235 were female and 171 were male. The mean age of participants was 17.51 with a standard deviation of ±1.42. The largest proportion of respondents was from Amhara ethnicity 401(98.8%) and among the study participants, 394(97%) were Orthodox religious followers. Of the respondents, 319(78.6%) were living with both parents. The majority of study participants 120(29.6%) were from grade 10. More than half of the study participants 264(65%) were originally from urban (Table 1).

**Table 1:** sociodemographic information on the research participants in 2021 in Northwest Ethiopia

Variable	Category	Frequency	Percentage
Age	14-16	30	7.4
	17-19	339	83.5
	≥20	37	9.1
Sex	Male	235	57.9
	Female	171	42.1
Religion	Orthodox	394	97
	Other*	12	3
Place of origin	Urban	264	65
	Rural	142	35
Living situation	Both parents	139	78.6
	Single parents	50	12.3
	Other**	37	9.1
Ethnicity	Amhara	401	98.8
	Other***	5	1.2
Academic performance	50-74.9	177	43.6
	75-84.9	134	32
	≥85	95	23.4
Class	Nine	105	25.9
	Ten	120	29.6
	Eleventh	94	23.2
	Thualeve	87	21.4
Father educational level	No formal education	69	17
	Educated	139	34.2
	Lernt 1-8	49	12.1
	Lernt 9-12	43	10.6
	Diploma	14	3.4
	Degree and more	92	22.7
Mother educational level	No formal education	117	28.8
	Educated	123	30.3
	Lernt 1-8	31	7.6
	Lernt 9-12	48	11.8
	Diploma	40	9.9
	Degree and more	47	11.6
Monthly pocket money	≤250	316	77.8
	251-500	55	13.5
	501-750	10	2.5
	751-1000	10	2.5
	>1000	15	3.7

* : muslim, catholic, and protestant; **: live with other relatives; ***: Oromo, Tigira and Guraga

**Psychological and clinical factors:** among the respondents 137(33.7%) had poor social support, 158(38.9%) had moderate social support and 111(27.4%) had strong social support. Of a total of study participants, 52(12.8%) had physical sexual abuse and 73(18%) had non-physical sexual abuse. Of the study participants, 66(16.3%) were influenced by peer pressure. Among the study participants, 72(17.7%) were doing physical exercise. Of the respondents, 119(29.3%) had common mental disorders 38(9.4%) had family exposure to mental illness and 84(20.7%) had family exposure to substance use (Table 2).

**Table 2:** psychological and clinical characteristics, Northwest Ethiopian high school students in 2021

Variables	Category	Frequency	Percentage
Social support	High	137	33.7
	Moderate	158	38.9
	Low	111	27.4
Sexual abuse	Abuse	52	12.8
	Non-abuse	354	87.2
Common mental disorder	Yes	119	29.3
	No	287	70.7
Peer pressure	Yes	66	16.3
	No	340	83.7

**Prevalence of substance use:** the current prevalence of substance use among study participants in Northwest Ethiopia was 213(52.5%). The prevalence of ever-use and current use of alcohol was 256(63.1%) and 199(49%) respectively. The prevalence of ever and current uses of khat were 165(40.6%) and 101(24.9%) respectively. The prevalence of ever and current use of tobacco was 97(23.9%) and 66(16.3%) respectively.

**Factors associated with the use of substances:** in univariable analysis being male, age, class level, poor social support, family history of substance use, academic performance, and the money given monthly. Among the factors, the probability of being male was 1.77 times the high risk to have substance use as compared to female students aOR: 1.77, 95% CI 1.11-2.77. Students who had a family exposure to substance use had 3.07 times high risk as compared to students who had no family exposure to substance use aOR: 3.07, 95% CI 1.57-6.01. The odds of developing substance use were 5.77 times riskier for students who had the effect of peer pressure as compared to students who had no effect of peer pressure aOR: 5.77, CI 95% 2.39-13.89 (Table 3).

**Table 3:** bi-variable and multi-variable logistic regression factors relating to substance use among high school students

Variables	Category	Substance use		COR with 95%CI	AOR with 95% CI
		Yes	No		
Sex	Female	108	127	1	1
	Male	105	66	1.87(1.25-2.80)	1.77(1.11-2.77) *
Age	14-16	20	10	1	1
	17-19	175	164	0.53(0.24-1.17)	0.57(0.23-1.39)
	≥20	18	19	0.47(0.18-1.28)	0.49(0.15-1.64)
Peer pressure	Yes	59	7	0.10(0.04-0.22)	5.77(2.39-13.89) *
	No	154	186	1	1
Academic performance	50-74.9	90	87	0.75(0.46-1.24)	0.91(0.51-1.60)
	75-84.9	68	66	0.75 (0.44-1.27)	0.74(0.40-1.33)
	>85	55	40	1	1
Family history of substance use	Yes	69	15	5.69(3.12-10.36)	3.07(1.57-6.01) *
	No	144	178	1	1
Social support	Poor	65	72	0.71(0.43-1.18)	0.79(0.45-1.40)
	Moderate	86	72	0.94(0.58-1.54)	0.95(0.55-1.64)
	Strong	62	49	1	1
Class	9	64	41	1	1
	10	67	53	0.81(0.48-1.38)	1.05(0.57-1.94)
	11	45	49	0.59(0.36-1.03)	0.80(0.41-1.55)
	12	37	50	0.47(0.27-0.85)	0.61(0.30-1.24)
Monthly given money	≤250	133	133	1	1
	251-500	34	20	1.70(0.93-3.10)	1.76(0.92-3.38)
	501-750	7	6	1.17(0.38-3.56)	0.90(0.23-3.59)
	751-1000	14	17	0.82(0.39-1.74)	0.71(0.17-2.99)
	>1000	25	17	1.47(0.76-2.85)	3.09(0.86-11.14)

* : significantly associated factors

## Discussion

The main objectives of this study were to determine the prevalence and to identify the associated factors associated with substance use. The overall current prevalence of substance use among the students who were involved in this study was 213 (52.5%) with (95% CI; 47.58-57.34). From this result, alcohol was the most consumed substance followed by khat and tobacco both in the lifetime and current use, other drugs other than these three were not used at all in this study. This finding was simultaneous with studies conducted in Northwest Ethiopia 47.9% [6]. The current prevalence is high as compared to other studies employed in Iran at 13.86% [7], and Rwanda at 34% [24]. This could be the result of cultural influence to drink alcohol especially (Tela) and the effect of high social interaction in Ethiopia that best friend influence to use the substance in this study. Of the study participant, 97% were orthodox religious followers drinking alcohol was considered and habit that increase the conception of this substance.

The current prevalence was lower than other studies carried out on high school students in Kenya 69.8% [25] and Francisco 69% [26]. In another way, this result is also lower as compared to studies conducted among university students in Haramaya and Jimma 62.4% and 68.2% respectively [27,28]. This might be due to the high effect of friend influence and stress related to their educations. From the respondent, the current use of substances varies due to cultural and other perspectives like the use of alcohol, tobacco, and khat were 49%, 16.3%, and 24.9% respectively. Among this study's participants, approximately three fourth of students 74.6% used at least one of the three substances in their lifetime but the most consumed substance was alcohol followed by khat.

The study indicated that there was a high prevalence of substance use in males than in women. The consumption of all substances which were used in this area becomes high in male students than females. Our finding was consistent with other studies done in Iran [7], and South Africa [29]. The possible reason for the high prevalence in males was due to cultural permission [7] and the role of gender-related to the external influence and the effect of sensation-seeking behaviors [6]. The consumption of those substances predominantly male practices is not acceptable among females if it´s used by a female they are considered sex workers [30]. The protective effect of the female gender is varying from country to country for instance the significant protective effect of gender in South Africa but not in the United States [29]. In this study area females were under social restrictions and controlled by their family members especially young and unmarried are restricted more than males [14].

The use of alcohol and other substance in family members or households was the risk to use of similar or other substances by siblings or students. This finding was supported by other two studies employed in Southeast and Northwest Ethiopia [6,9]. This might be because of learned behavior among children for the consumption of substances [6]. The use of substances by family members is more likely to increase substance use behavior due to the familiarization of substances thereby decreasing the social norm perceived perception of students [31]. Family substance abuse influences adolescent drug use and psychologically influences children to use that drug as learned from parents [32].

Having a best friend who used substances three times made the respondent use any of the substances when compared with other students who had no best friends who consumed substances. This finding is also congruent with other findings carried out in Ethiopia [6,9,25] and South Africa [29]. This could be due to the impact of social norms and the learned behavior of students from their peers was considered important, particularly among students [6,25]. In this study area, the consumption of those substances was considered a sign of intelligence that influence other students to use substances. Most participants showed that they used those substances for relaxation and to relieve the stress related to their education to feel good [25]. The use of some substances was for study to increase concentration and to decrease the sleep duration when students study their course in the group, particularly khat. Most likely this has occurred on the weekends when they interact with colleagues, which could result in the use of many substances at once or the combining of various drugs [33]. The power of the friend use and pressure to use substances and the availability that particular substances which consumed by those friends had a great social influence for the use of substance [34].

**Limitations of the study:** even though this research has great strengths like the use of standardized screening tools and determining the burden of substance use but it has also its weakness. The nature of the cross-sectional study can´t point out the temporal relations of the outcome variables and the factors associated with them.

## Conclusion

The prevalence of substance use among high school students was high as compared to other studies. Being male, family history of substance use, and having peer pressures were strongly associated with substance use. Therefore, to decrease the prevalence of substance use among students the associated factors should be controlled and minimized.

### What is known about this topic

- *The majority of the worldwide burden of disease is associated with the chronic use of the substance;*
- *The most consumed substances were found alcohol, cigarettes, and khat, particularly in Ethiopia;*
- *The consumptions of substances have a negative impact on the academic performance of high school students*.

### What this study adds

- *The current prevalence of substance use among high school students was 52.5% which is more than half;*
- *The consumption of alcohol is by far higher than other substances among students with a prevalence of 49%;*
- *The presence of peer pressure to use substances was the most highly affected student which means 5.77 times more than other students*.
